# Deep learning in computed tomography to predict endotype in chronic rhinosinusitis with nasal polyps

**DOI:** 10.1186/s12880-024-01203-w

**Published:** 2024-01-24

**Authors:** Weidong Du, Weipiao Kang, Shixin Lai, Zehong Cai, Yaowen Chen, Xiaolei Zhang, Yu Lin

**Affiliations:** 1 Department of Otolaryngology-Head and Neck Surgery, The Second Affiliated Hospital of Shantou University Medical College, 69 North Dongxia Road, 515041 Shantou, Guangdong China; 2 College of Engineering, Shantou University, 515063 Shantou, China; 3 Department of Radiology, The Second Affiliated Hospital of Shantou University Medical College, 69 North Dongxia Road, 515041 Shantou, Guangdong China

**Keywords:** Chronic rhinosinusitis with nasal polyps, Deep learning, Endotype, Precision treatment, Sinus computed tomography

## Abstract

**Background:**

As treatment strategies differ according to endotype, rhinologists must accurately determine the endotype in patients affected by chronic rhinosinusitis with nasal polyps (CRSwNP) for the appropriate management. In this study, we aim to construct a novel deep learning model using paranasal sinus computed tomography (CT) to predict the endotype in patients with CRSwNP.

**Methods:**

We included patients diagnosed with CRSwNP between January 1, 2020, and April 31, 2023. The endotype of patients with CRSwNP in this study was classified as eosinophilic or non-eosinophilic. Sinus CT images (29,993 images) were retrospectively collected, including the axial, coronal, and sagittal planes, and randomly divided into training, validation, and testing sets. A residual network-18 was used to construct the deep learning model based on these images. Loss functions, accuracy functions, confusion matrices, and receiver operating characteristic curves were used to assess the predictive performance of the model. Gradient-weighted class activation mapping was performed to visualize and interpret the operating principles of the model.

**Results:**

Among 251 included patients, 86 and 165 had eosinophilic or non-eosinophilic CRSwNP, respectively. The median (interquartile range) patient age was 49 years (37–58 years), and 153 (61.0%) were male. The deep learning model showed good discriminative performance in the training and validation sets, with areas under the curves of 0.993 and 0.966, respectively. To confirm the model generalizability, the receiver operating characteristic curve in the testing set showed good discriminative performance, with an area under the curve of 0.963. The Kappa scores of the confusion matrices in the training, validation, and testing sets were 0.985, 0.928, and 0.922, respectively. Finally, the constructed deep learning model was used to predict the endotype of all patients, resulting in an area under the curve of 0.962.

**Conclusions:**

The deep learning model developed in this study may provide a novel noninvasive method for rhinologists to evaluate endotypes in patients with CRSwNP and help develop precise treatment strategies.

## Background

Chronic rhinosinusitis (CRS) is a chronic inflammatory disease of the upper respiratory tract with high incidence (8%), recurrence rate (50%), and medical costs (33 billion dollars per year) [1–4]. Recently, as the pathophysiological mechanisms of CRS continue to be revealed, the concept of “endotype” has been proposed, which is a crucial driving factor for precise treatment in patients with CRS. However, as yet, no unified classification standard for CRS endotypes has been proposed, and clinical guidelines often classify CRS as either eosinophilic CRS (eCRS) or non-eosinophilic CRS (non-eCRS) [5, 6].

Different CRS endotypes require different clinical treatment strategies. As for pharmacotherapy, corticosteroid and doxycycline have been shown to effectively treat eosinophilic CRS with nasal polyps (eCRSwNP), whereas macrolide antibiotics address non-eosinophilic CRSwNP (non-eCRSwNP). A surgical approach aimed to preserve the nasal sinus mucosa is more suitable for non-eCRSwNP rather than eCRSwNP [5–8]. Thus, the timely diagnosis of these endotypes is crucial for the development of precise treatment strategies.

Biopsy is the “gold standard” for diagnosing endotypes. However, preoperative biopsy cannot reflect the overall inflammation of the disease and has a certain misdiagnosis rate, whereas postoperative biopsy can only clarify the endotype after surgery, missing precise preoperative medication treatments. Therefore, the preoperative identification of endotype is an urgent issue.

Prediction models, which serve as a new noninvasive diagnostic method, play an important role in addressing this challenge. Sinus computed tomography (CT) is the routine imaging examination used for CRS diagnosis and treatment planning, and CT data are often used to construct predictive models [9]. However, the Lund-Mackay (LM) scoring scale, global osteitis scoring scale (GOSS), or radiomics methods used in previous studies to quantify sinus CT data are all shallow prediction models and cannot overcome the subjective interference caused by manual extraction of the image features [8, 10–13]. Emerging artificial intelligence methods, especially deep learning (DL), not only automatically segment and extract image features but also analyze the high-dimensional pivotal features of images, making the results simpler and more reliable.

One DL algorithm based on sinus CT to predict the endotype in patients with CRS has been proposed; however, there remain issues with the algorithms, data volume, and grouping, which limit its overall predictive performance [14]. Therefore, the present study aims to construct a novel DL-based model for predicting the endotypes of CRSwNP based on sinus CT data, thus providing a new noninvasive method for the diagnosis of the endotype and developing new ideas for the precise treatment of patients with CRSwNP.

## Methods

### Study design and population

This retrospective study was conducted in a single tertiary teaching hospital and analyzed the clinical characteristics of patients with CRSwNP in Shantou City, Guangdong Province, China. The clinical data were extracted from electronic medical records using a standard Excel form at the Second Affiliated Hospital of Shantou University Medical College between January 1, 2020, and April 31, 2023, and included patient demographic characteristics, disease history, clinical features, images, and laboratory results. Patients were identified by our clinical rhinologists using the diagnostic criteria for CRSwNP recommended by the European Position Paper on Rhinosinusitis and Nasal Polyp 2020, and none of the patients had been treated with antibiotics or corticosteroids within 4 weeks before surgery [5, 6]. Patients with the following conditions were excluded: (1) CRS without nasal polyps, (2) age < 18 years, (3) fungal sinusitis, (4) posterior nostril polyp, (5) benign or malignant nasal and sinus tumors, (6) cystic fibrosis, (7) primary ciliary dyskinesia, and (8) no sinus CT examination and functional endoscopic sinus surgery (FESS). Two rhinologists independently checked the records. This study was approved by the Ethics Committee of the Second Affiliated Hospital of Shantou University Medical College, which waived the requirement for written informed consent owing to the use of deidentified retrospective data.

### Quality control for sinus CT images

The patients underwent paranasal sinus scanning with 64-slice spiral CT using a tube voltage of 120 kV and a tube current of 200 mA. CT images were obtained with 0.5–0.6-mm collimation and reconstructed into axial, coronal, and sagittal images every 1.0 mm on a 512 × 512 matrix using iterative reconstruction algorithms associated with each vendor’s CT scanner. All CT image data for each patient, including the axial, coronal, and sagittal planes, were downloaded from the picture archiving and communication system (PACS) and saved in the Digital Imaging and Communications in Medicine (DICOM) format. To establish the most direct and effective method of satisfying the DL model, the original DICOM files were converted to intensity data (PNG format).

### Diagnosis of the endotype

**Fig. 1:**
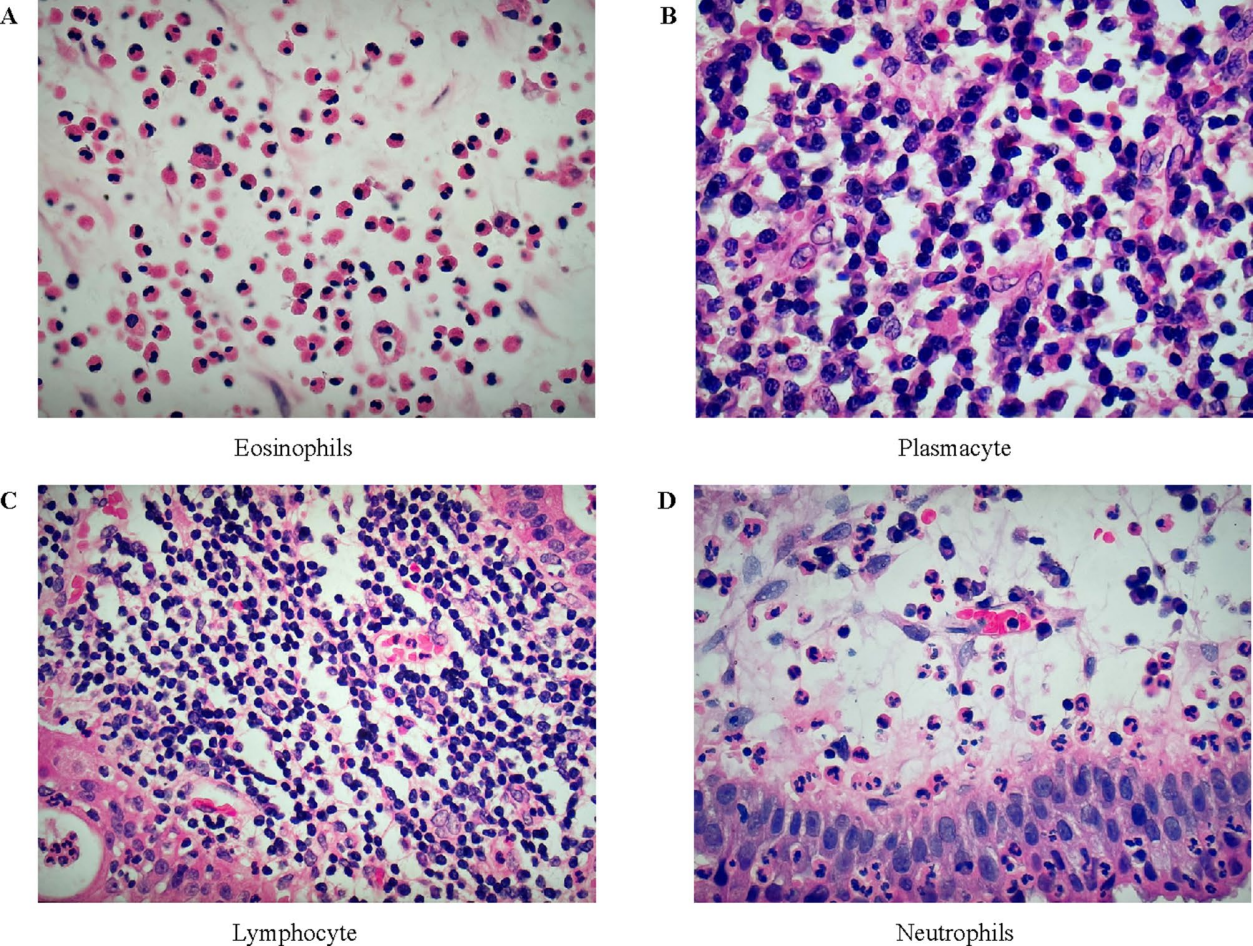
Inflammatory cells in nasal polyp tissue (H-E staining) under high-power microscopy (× 400). (**A**, **B**, **C**, and **D**) represent the count of each inflammatory cell in a high-power field of view, respectively. H-E, hematoxylin and eosin

Intraoperative nasal polyps were processed using routine hematoxylin and eosin staining. All stained samples were observed by two independent pathologists using a bright-field light microscope (BX51; Olympus, Tokyo, Japan) at ×400 magnification. Ten randomly selected non-overlapping fields were observed, and the mean counts of eosinophils and inflammatory cells (including plasmacytes, lymphocytes, and neutrophils) were calculated **(**Fig. 1**)**. eCRSwNP was diagnosed as the presence of > 10% tissue eosinophils among the total inflammatory cells, as previously described [15].

### Residual networks and dataset construction

**Fig. 2:**
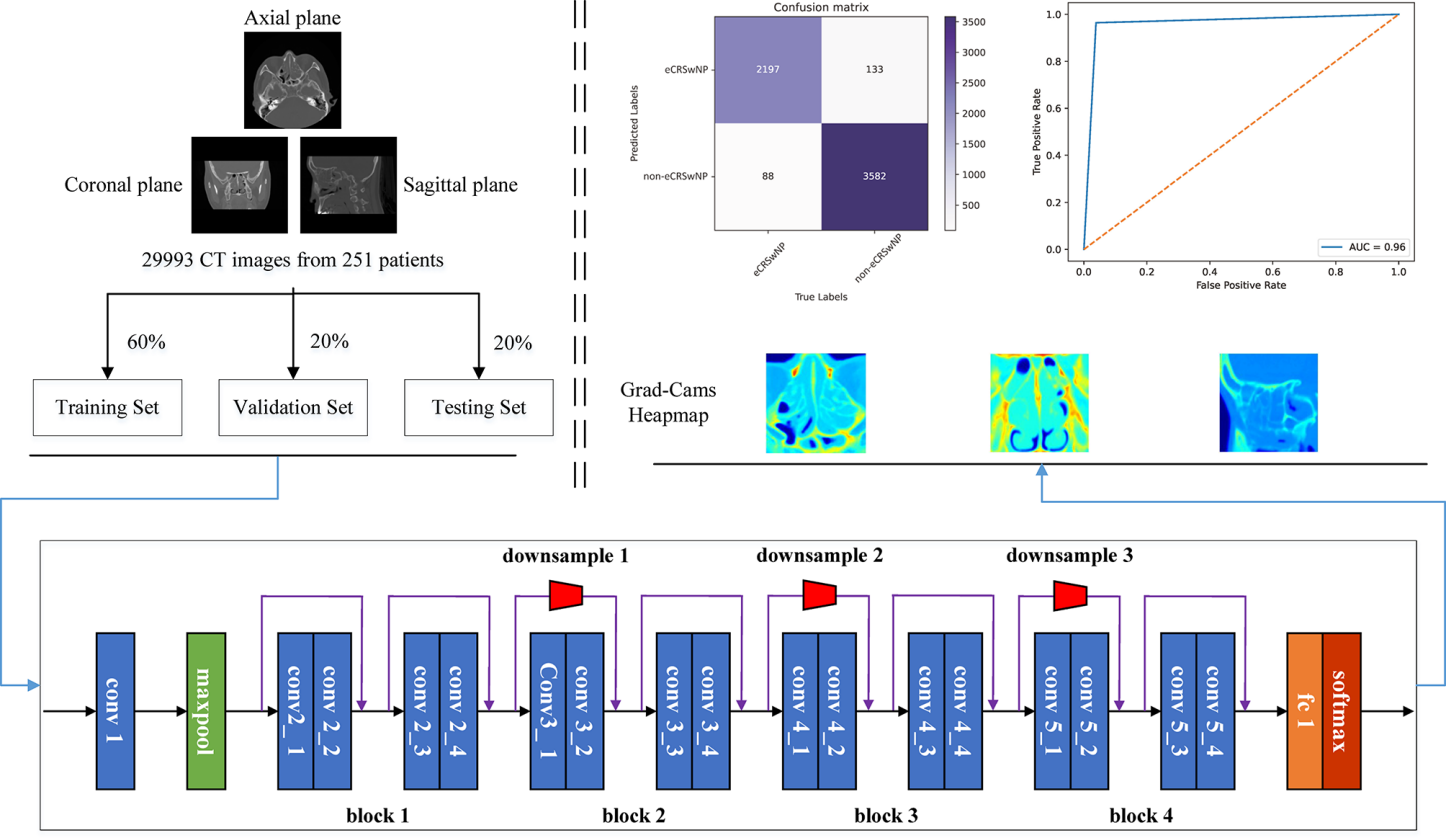
The operating principle of the ResNet-18 for predicting the endotype in patients with CRSwNP. CRSwNP, chronic rhinosinusitis with nasal polyps; ResNet-18, residual network-18

The Residual Network (ResNet) framework solves degradation problems by constructing a network using modules called residual models. Compared with deep neural networks, the ResNet framework can improve training and gain accuracy from a considerably increased depth. We used ResNet-18 to perform the classification task [16], as shown in Fig. 2.

The dataset for ResNet-18 was randomly divided into training, validation, and testing sets in a ratio of 6:2:2. Briefly, the training set was used to train our network model and determine the parameters. The validation set was used to modify the network structure and adjust the hyperparameters, and the test set was used to verify the generalization ability of our model. The predicted data were compared with ground truth data to evaluate the performance of the proposed network model. The experiments were performed using TensorFlow on a workstation with an Intel Core i5-12400f CPU and NVIDIA GeForce RTX 3060 GPU. Our model used twenty epochs and 20 batchsize using the Adam optimizer, a learning rate of 0.0002, the beta1 and beta2 parameters were adjusted to 0.9 and 0.999, respectively, during the training process.

### Statistical analysis

Continuous variables are presented as medians and interquartile ranges (IQRs), while categorical variables are presented as frequencies and percentages (%). Intergroup differences were assessed using the Mann–Whitney U, chi-square, and Fisher’s exact tests.

The accuracy, recall, precision, confusion matrix, receiver operating characteristic (ROC) curve, and area under the ROC curve (AUC) were used as performance metrics to evaluate the classification performance of our model. This classification produces four parameter types: true positives (TP), true negatives (TN), false positives (FP), and false negatives (FN). The accuracy was calculated as the ratio between the number of correctly predicted samples to the total number of samples. Recall was calculated as the ratio between the predicted number of TP and the total number of positive predicted samples. Precision was calculated as the ratio between the predicted number of TP and the actual total number of positive samples. The confusion matrix conveyed a 2 × 2 table formulated with these four outcomes. The ROC curves explored the effects on the true positive rate (TPR) and the false positive rate (FPR) as the position of an arbitrary decision threshold was varied, with larger AUCs indicating more accurate recognition. The formulas for accuracy, recall, and precision are presented in Eqs. (1, 2), respectively.

1 $$Accuracy=\frac{{TP+TN}}{{TP+TN+FP+FN}},$$

2 $$Precision\,=\,\frac{{TP}}{{TP\,{\text{+}}\,FP}},\,Recall\,=\,\frac{{TP}}{{TP\,{\text{+}}\,{\text{FN}}}},$$

Gradient-weighted class activation mapping (Grad-Cam) was performed by extracting feature maps from the final layer of ResNet-18 to interpret the model. Cohen’s kappa scores were calculated to interpret the consistency between the predicted and true labels in the confusion matrix. Statistical analyses were performed using Python software (Python Software Foundation, version 3.9.0) and R environment, version 4.3.1 (R Foundation for Statistical Computing, Vienna, Austria). All statistical tests were two-tailed, and *P* <.05 was considered significant in each statistical analysis.

## Results

### Baseline characteristics

Based on the inclusion and exclusion criteria, this study included 251 patients with CRSwNP. The median patient age was 49 years (IQR 37–58 years) and 153 (61.0%) were male. The median disease duration was 12.0 months (IQR 3.0–60.0 months). Twelve (4.8%) and 41 (16.3%) patients had diabetes mellitus and hypertension, respectively. Smoking and drinking histories were observed in 40 (15.9%) and 21 (8.4%) patients, respectively. The confounding factors, such as age, sex, disease duration, smoking, alcoholism, diabetes mellitus, and hypertension, were not significantly different between the eCRSwNP and the non-eCRSwNP groups **(**Table 1**)**, which demonstrated that these potential confounding factors between the two groups have been effectively controlled, thus reducing the impact on the bias of the results.

**Table 1:** Demographics and clinical characteristics of patients with chronic rhinosinusitis with nasal polyps

Characteristics	All (n = 251)	e-CRSwNP (n = 86)	Non- eCRSwNP (n = 165)	P
Age (years), median (IQR)	49.0 (37.0–58.0)	48.0 (41.0–54.75)	50.0 (32.0–58.0)	0.838
Sex, male, n (%)	153 (61.0)	53 (61.6)	100 (60.6)	0.983
Disease duration (months), median (IQR)	12.0 (3.0–60.0)	12.0 (4.25–60.0)	12.0 (3.0–48.0)	0.574
Smoking, n (%)	40 (15.9)	18 (20.9)	22 (13.3)	0.168
Alcoholism, n (%)	21 (8.4)	11 (12.8)	10 (6.1)	0.112
Diabetes mellitus, n (%)	12 (4.8)	2 (2.3)	10 (6.1)	0.315
Hypertension, n (%)	41 (16.3)	12 (14.0)	29 (17.6)	0.578
Number of sinus CT images	29,993	11,019	18,974	-
Axial plane, n (%)	10,651 (35.5)	3772 (34.2)	6879 (36.6)	**-**
Coronal plane, n (%)	9767 (32.5)	3592 (32.6)	6175 (32.4)	**-**
Sagittal plane, n (%)	9575 (32.0)	3655 (33.2)	5920 (31.2)	**-**

Data are presented as median and IQR for continuous variables and numbers with percentage for categorical variables, were analyzed by Mann-Whitney U-test. Abbreviation: CRSwNP, chronic rhinosinusitis with nasal polyps; IQR, interquartile range; e-CRSwNP, eosinophilic CRSwNP; Non- eCRSwNP, non-eosinophilic CRSwNP; CT, computed tomography

### Summary of the sinus CT images

In this study, 11,019 sinus CT images (axial plane, 3,772 images; coronal plane, 3,592 images; sagittal plane, 3,655 images) were obtained from 86 (34.3%) patients diagnosed with e-CRSwNP. Likewise, 18,974 sinus CT images (axial plane, 6,879 images; coronal plane, 6,175 images; sagittal plane, 5,920 images) were obtained from the remaining 165 (65.7%) patients diagnosed with non-eCRSwNP. The sinus CT images were randomly split into the training (17,995 images), validation (5,998 images), and testing sets (6,000 images).

### Performance of the DL model based on sinus CT data

**Fig. 3:**
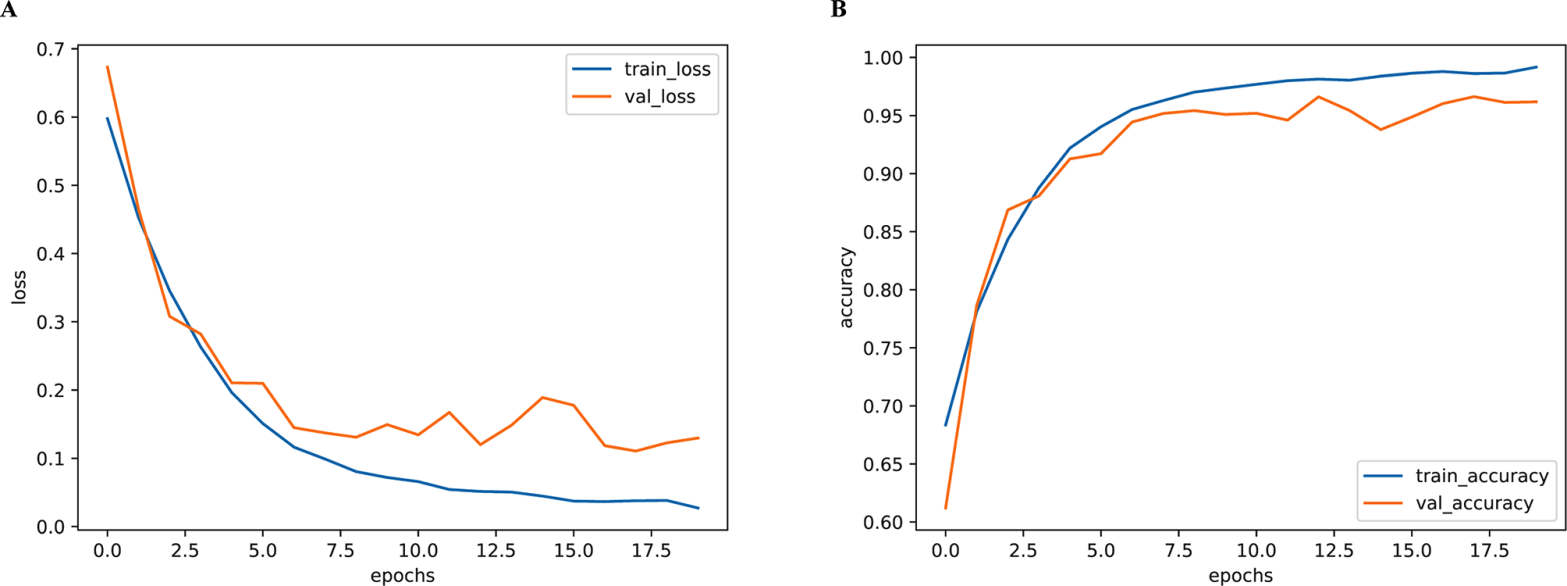
Model loss (**A**) and accuracy (**B**) curves over epochs in the training and validation datasets

The loss and accuracy curves of our model over the epochs in the training and validation sets of the wake vortex dataset are shown in Fig. 3. The loss and accuracy obtained by our model tended to converge quickly and reach a steady state.

**Fig. 4:**
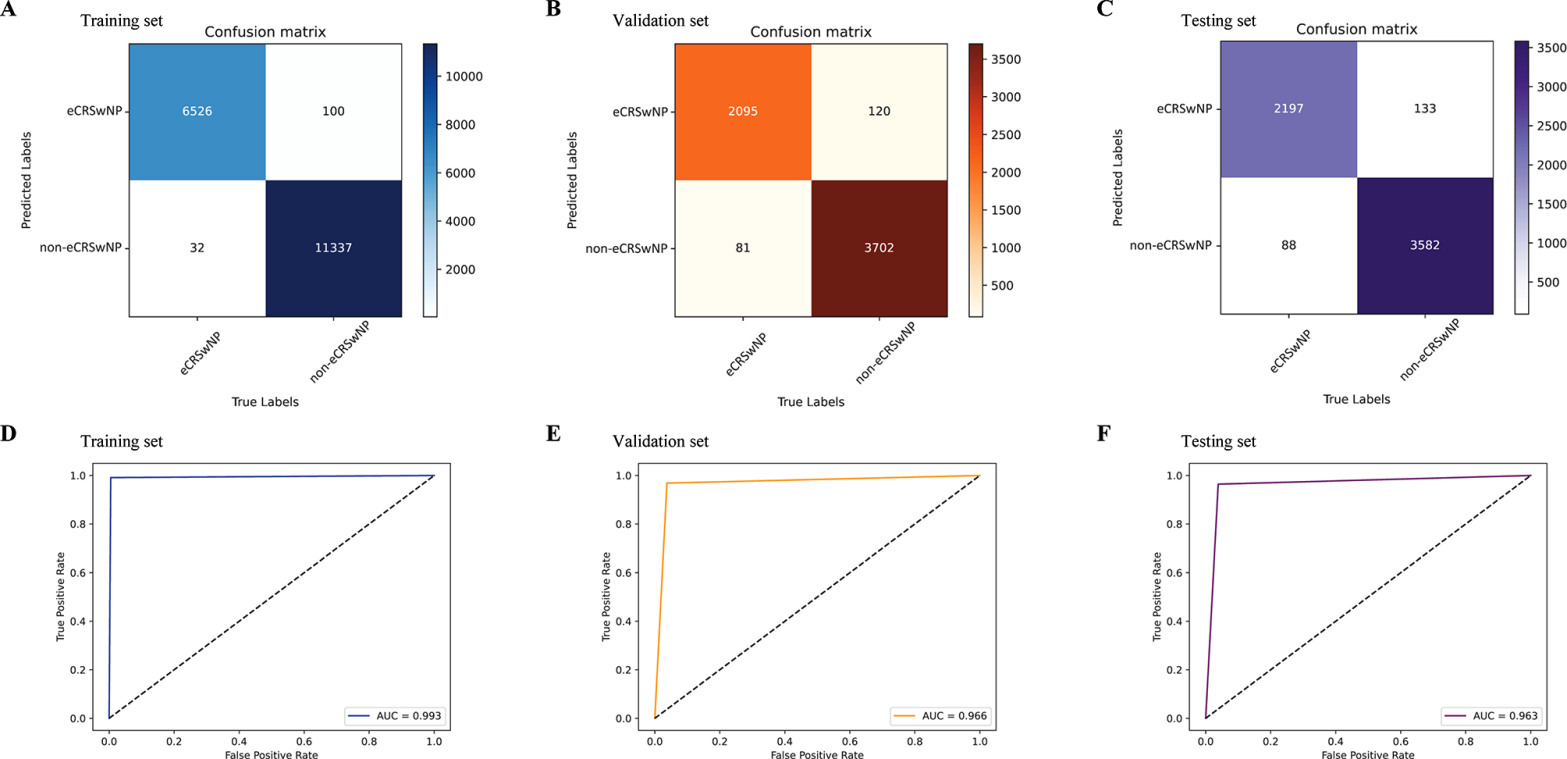
Confusion matrixes and ROCs of ResNet-18. (**A**, **D**) Training dataset. (**B**, **E**) Validation dataset. (**C**, **F**) Training dataset. ROC, receiver operating characteristic; ResNet-18, residual network-18

We first created a confusion matrix by assigning the predicted and true labels to the x- and y-axes, respectively **(**Fig. 4A–C**)**. In the training set, the kappa score was 0.985 (95% confidence interval [CI] 0.982–0.987), and the DL model had good discriminative performance, with an AUC of 0.993 (Fig. 4D). Further clarification of the predictive performance of the DL model in the validation set showed the accurate prediction of 2,215 eCRSwNP and 3783 non-eCRSwNP images among 5,998 total images (kappa = 0.928; 95% 0.918–0.938) **(**Fig. 4B**)**, with an AUC of 0.966 **(**Fig. 4E**)**. An additional classification experiment in the testing set was performed to assess the generalizability of the DL model, the confusion matrix of which showed good consistency (kappa = 0.922; 95%CI 0.912–0.932). Figure 4F shows the ROC curves obtained from the testing set, which also showed good discriminative performance (AUC = 0.963). Table 2 presents the accuracy, F1, sensitivity, and specificity of the DL model in the training, validation, and testing sets.

**Table 2:** Predictive performance of the deep learning model in the training, validation, and testing sets

DataDatasets	Precision	F1	Sensitivity	Specificity
Training set	0.985	0.990	0.995	0.991
Validation set	0.946	0.954	0.963	0.969
Testing set	0.943	0.952	0.961	0.964

We then used the constructed DL model to predict all patients with CRSwNP, which showed an AUC of 0.962. The predictive performance of the DL model in eCRSwNP and non-eCRSwNP patients was excellent, with AUCs of 0.960 and 0.964, respectively.

### DL model interpretability

As shown in Fig. 2, Grad-Cams were obtained from the final layer of the neural network. The yellow and red highlights in the heatmap correlate with the classification. The area enclosed by these highlights was the sinus region, which indicated the accurate positioning of the model when interpreting the images.

## Discussion

Clarifying the endotype is a prerequisite for implementing precise treatment for patients with CRSwNP. To our knowledge, this is the first DL model constructed using ResNet-18 to predict the CRSwNP endotype. Moreover, the data width of CT images was expanded to the three-dimensional level (axial, coronal, and sagittal planes). Our DL model showed good predictive performance and generalizability in the training, validation, and testing sets. This result provides a new noninvasive diagnostic method for rhinologists to classify endotypes in patients with CRSwNP, guide the development of personalized and precise treatment plans, and provide a reference basis for prognosis evaluation.

Currently, the LM and GOSS scoring scales are the most commonly used tools to quantify the CT image features of the sinuses; therefore, these two tools are often used to predict CRSwNP endotypes. Meng et al. used the LM scoring scale to predict the CRSwNP endotype, in which the ethmoid and maxillary sinuses (E/M ratio) had the highest predictive value, with an AUC of 0.938 (sensitivity, 94.2%; specificity, 89.6%) [10]. Other studies on prediction models for CRS endotypes based on the GOSS reported that an ethmoid osteitis score > 4.5 (AUC, 0.690; sensitivity, 62.0%; specificity, 71.0%) may be used as a predictor for patients with eCRS [11, 12]. Although both tools have a certain predictive performance, they use semi-qualitative and semi-quantitative data, and the results are unavoidably influenced by subjective factors. Furthermore, the data statistics in these tools consume considerable time and waste resources (manpower, material, and financial resources); thus, they are not suitable in the current era of big data.

To improve the time-consuming and labor-intensive challenges of these assessments, radiomics is a new and effective way to predict the CRSwNP endotype. A recent study reported good performance in predicting the endotype of CRSwNP using radiomics based on sinus CT data, with an AUC of 0.815 [13]. However, radiomics requires manual interventions for both image segmentation and feature extraction, indicating that its working principle cannot be separated from the essence of semi-qualitative and semi-quantitative data [17, 18]. Moreover, the results are affected by subjective factors, resulting in poor prediction performance.

Emerging DL technology such as ResNet-18 has powerful automatic image segmentation and feature extraction capacity and is especially adept at automatically capturing key high-dimensional image features in complex images, which omits the manual intervention stage and allows more objective and realistic prediction results [19, 20]. Therefore, DL appears to be more efficient and accurate than radiomics. Hua et al. constructed an endotype prediction model for CRSwNP based on sinus CT data using U-net and Deeplabv3 neural networks, which resulted in an AUC between 0.80 and 0.90 for both predicting images and patients [14]. However, their study was limited by the depth of the neural networks and the width of the single-channel data (only data on the axial plane of the sinus CT images), which did not achieve high-accuracy prediction performance in the case of image annotation.

The DL model constructed in this study not only solves the above-mentioned time- and labor-intensive problems but also eliminates the challenge of subjective interference caused by human intervention during automatic image segmentation and feature extraction. More importantly, the DL model addresses the issues of data depth and width to achieve a new level of predictive performance (AUC = 0.993). In addition, we confirmed the effectiveness and generalizability of this model through validation (AUC = 0.966) and testing (AUC = 0.963). This model can provide a noninvasive method for rhinologists to diagnose the endotypes in patients with CRSwNP before starting treatment, thereby guiding the development of personalized and precise treatment plans [5–8]. If a patient with CRSwNP is predicted to have eCRSwNP, an oral corticosteroid can be considered; surgery can be performed by extended endoscopic sinus surgery or reboot surgery, and the model can predict that such patients are prone to recurrence after surgery. If a patient with CRSwNP is predicted to have non-eCRSwNP, oral clarithromycin can be considered; the nasal sinus mucosa should be preserved as much as possible during FESS, and the recurrence rate in such patients is predicted to be low after the operation.

### Limitations

This study has several limitations. First, compared to patients with chronic rhinosinusitis without nasal polyps (CRSsNP), those with CRSwNP have higher rates of postoperative recurrence and conservative drug treatment failure. Therefore, patients with CRSwNP are attracting increasing attention from rhinologists in clinical practice. Accordingly, the main focus of this study was on patients with CRSwNP; therefore, this DL model is not applicable to the patients with CRSsNP. Second, this study had limitations inherent to the retrospective design, including selection and recall biases. Therefore, the predictive performance of this model must be further validated using prospective data before it can be used clinically. Third, this was a single-center study, with each patient in each region having their own regional characteristics; therefore, the model constructed in this study may not be applicable to patients in other areas. We will further upgrade the generalizability of the model in the future by integrating sinus CT data from multiple centers to iteratively update the model. Fourth, ResNet-18 has powerful capabilities for automatic image segmentation and feature extraction, which can solve the problems of gradient vanishing and explosion in network architectures, thereby supporting deeper architectures and reducing overfitting. However, it resembles a “black box” with poor interpretability, which is a common problem in most artificial intelligence algorithms [21, 22]. Although this study achieved good predictive performance without annotations, the model interpretability was poor, as the highlights in the heatmap mainly focused on the bone rather than on the polyps. In fact, sinus osteitis is also an evaluation indicator for predicting the endotype, but this study would prefer to obtain high-dimensional imaging features of the polyp tissue to better interpret the effectiveness of the model. Thus, enhancing the interpretability and transparency of the DL model is a future research direction and is also the driving force for its continuous iteration and updating.

## Conclusion

The results of this study provide a new noninvasive method for rhinologists to predict the endotype of patients with CRSwNP, which showed good predictive performance and generalizability. To promote the application of the DL model nationwide, sinus CT data should be prospectively collected from multiple centers to further verify the accuracy and generalizability of this DL model and continuously iterate and update the technical version of the model. The results of the present study will help rhinologists evaluate the endotypes in patients with CRSwNP before starting treatment, develop personalized and precise treatment strategies, and predict disease prognosis.

## Data Availability

The datasets used and/or analyzed in the current study are available from the corresponding author upon reasonable request.

## Abbreviations

AUC: area under the ROC curve
CI: confidence interval
CRS: chronic rhinosinusitis
CRSwNP: chronic rhinosinusitis with nasal polyps
CT: computed tomography
DICOM: Digital Imaging and Communications in Medicine
DL: deep learning
eCRS: eosinophilic CRS
eCRSwNP: eosinophilic CRSwNP
FESS: functional endoscopic sinus surgery
FP: false positives
FPR: false positive rate
FN: false negatives
GOSS: global osteitis scoring scale
Grad-Cam: Gradient-weighted class activation mapping
IQR: interquartile range
LM: Lund-Mackay
non-eCRS: non-eosinophilic CRS
non-eCRSwNP: non-eosinophilic CRSwNP
PACS: picture archiving and communication system
ResNet: Residual Network
ROC: receiver operating characteristic
TN: true negatives
TP: true positives
TPR: true positive rate
CRSsNP: chronic rhinosinusitis without nasal polyps
